# FEB-YOLOv8: A multi-scale lightweight detection model for underwater object detection

**DOI:** 10.1371/journal.pone.0311173

**Published:** 2024-09-27

**Authors:** Yuyin Zhao, Fengjie Sun, Xuewen Wu

**Affiliations:** Department of Cyberspace Security, Hainan University, Haikou, Hainan Province, China; The University of Tokyo: Tokyo Daigaku, JAPAN

## Abstract

Underwater object detection plays a crucial role in safeguarding and exploiting marine resources effectively. Addressing the prevalent issues of limited storage capacity and inadequate computational power in underwater robots, this study proposes FEB-YOLOv8, a novel lightweight detection model. FEB-YOLOv8, rooted in the YOLOv8 framework, enhances the backbone network by refining the C2f module and introducing the innovative P-C2f module as a replacement. To compensate for any potential reduction in detection accuracy resulting from these modifications, the EMA module is incorporated. This module augments the network’s focus on multi-scale information, thus boosting its feature extraction capabilities. Furthermore, inspired by Bi-FPN concepts, a new feature pyramid network structure is devised, achieving an optimal balance between model lightness and detection precision. The experimental results on the underwater datasets DUO and URPC2020 reveal that our FEB-YOLOv8 model enhances the mAP by 1.2% and 1.3% compared to the baseline model, respectively. Moreover, the model’s GFLOPs and parameters are lowered to 6.2G and 1.64M, respectively, marking a 24.39% and 45.51% decrease from the baseline model. These experiments validate that FEB-YOLOv8, by harmonizing lightness with accuracy, presents an advantageous solution for underwater object detection tasks.

## 1. Introduction

As the largest resource treasure house on earth, the ocean contains many valuable resources that have not yet been utilized. In order to fully understand, utilize and protect these resources, scientific and effective ocean management is indispensable [1]. However, humankind’s understanding of the underwater world is still relatively limited, and exploring the seabed faces multiple challenges such as high pressure, darkness, and extreme temperature differences. In particular, the rapid attenuation of light in water has caused various problems in underwater imaging technology, such as blur, noise and image distortion [2]. Traditional ocean detection methods mainly rely on human divers. All underwater object detection and identification work needs to be completed by people themselves, which is very difficult.

The progression of computer vision technology has ushered in a new era for oceanic exploration, enabling the use of non-invasive underwater detection equipment to probe and harness oceanic ecological resources [3]. These cutting-edge technologies have found broad applications across numerous marine activities, such as aquaculture, deep-sea fishing, marine biodiversity monitoring, environmental conservation, and underwater archaeology [4]. They represent innovative and efficient tools for the development and sustainable management of marine resources [5].

The field of object detection technology has evolved significantly over the past two decades [6, 7]. The timeline of its development can be bifurcated at the year 2014, marking the transition from traditional to deep learning-based detection methods. Prior to 2014, traditional detection methods mainly used sliding window techniques to acquire candidate frames in an image, and subsequently extracted the information within these frames, a process that typically involves the identification of features such as colour, texture and shape, which are ultimately fed into pre-trained classifiers for recognition. Since this approach relies heavily on manual feature extraction, its robustness and accuracy are relatively limited, and representative techniques include Histogram of Oriented Gradient (HOG) [8], Deformable Part Model (DPM) [9], and AlexNet [10]. After 2014, a new chapter was opened by the rise of deep learning-based detection methods. These methods are mainly divided into two categories: two-stage target detection methods such as Single Shot MultiBox Detector (SSD) [11], Faster R-CNN [12], etc., which first need to select candidate frames and then classify them; and single-stage target detection methods such as You Only Look Once (YOLO) [13–21], which omit the candidate frame selection step and are able to classify and localise images directly. These deep learning-based methods successfully overcome many of the limitations of traditional techniques and significantly improve the performance of the detection task, making deep learning-based object detection a hot research topic in the field [22].

The optimal underwater object detection model should adeptly balance high accuracy, operational efficiency, and the capacity to adapt to the multifaceted nature of underwater environments [23]. However, several challenges impede the attainment of this ideal. These include:

Accurately detecting small and clustered underwater objects presents a considerable challenge. These objects are often found in close proximity and are small in size, which complicates feature extraction, leading to less-than-optimal detection performance by the model. This issue underscores the need for sophisticated methods that enhance the model’s capability to discern and accurately classify such intricate scenarios.

The extensive size and significant computational complexity of object detection models complicate deploying them on embedded devices commonly used in the field. Historically, underwater object detection technologies have focused on enhancing detection accuracy through the use of sophisticated and extensive detection networks. However, these advanced models require high-end hardware, which often surpasses the capabilities of standard underwater detection devices. This limitation poses a significant challenge for the practical deployment of these models [24].

In response to these challenges, various studies have been conducted. To address underwater image degradation, Liu et al. [25] introduced a groundbreaking plug-and-play lightweight module known as the UnitModule. This innovative addition has been shown to significantly ameliorate detection performance in underwater object detector models. Zhang et al. [26] proposed a novel lightweight network for underwater image enhancement. This network incorporates DepthSepConv into its backbone, to reduce convolution operations and speed up memory operations. They also incorporated fewer residual connections to minimize overhead and introduced the Squeeze-and-Excitation (SE) module to improve model detection performance. Guo et al. [27] optimized the YOLOv8s model, employing FasterNet as the new backbone network to reduce model complexity, replaced the last C2f of the backbone network with GSConv’s improved C2f, and modified the feature pyramid to simplify model complexity. This resulted in an improvement in detection speed. Zhang et al. [28] made notable contributions to underwater garbage detection by proposing a cutting-edge algorithm. This algorithm integrates the Contrastive Boundary-enhanced Block (CBeB) module into the structural framework of the backbone network, significantly enhancing the model’s feature recognition capabilities. Additionally, it incorporates the Content-Aware ReAssembly of FEatures (CARAFE) module as a novel approach to upsampling, and introduces both the Efficient Multi-Scale Attention (EMA) module and Biformer module, aimed at strengthening the network’s feature extraction efficiency. These pivotal enhancements have played a crucial role in significantly elevating the network’s detection accuracy, marking a substantial leap forward in the realm of underwater detection technologies.

Despite recent advancements, current underwater object detection algorithms still exhibit notable limitations. Although these networks improve accuracy, they often inadvertently increase model complexity, which necessitates sophisticated computer hardware. In the context of survey equipment, the substantial size and computational demands of these models significantly impede deployment. This situation underscores a critical and pressing challenge: Achieving an optimal balance between performance and resource consumption in resource-constrained environments and crafting a more streamlined network model tailored to specific operational needs. To address these issues, this paper introduces a novel underwater object detection algorithm named FEB-YOLOv8, which builds upon the foundation of YOLOv8. This model can better balance the relationship between model accuracy and complexity. While being lightweight, its accuracy is also improved. The contributions of this article are as follows:

1. The integration of Partial Convolution (PConv) from FasterNet [29] to refine the original C2F structure, introducing a P-C2F structure that minimizes redundant gradient computations and simplifies the network model.
2. The implementation of the EMA attention mechanism [30] into the backbone network significantly sharpens the model’s ability to concentrate on vital information. This strategy enhances feature extraction, which in turn substantially boosts the model’s detection capabilities.
3. Drawing on the ideas of Bidirectional Feature Pyramid Network (Bi-FPN) [31], a new feature pyramid network structure is designed, which improves model detection accuracy while being lightweight.

## 2. Related works

### 2.1 Introduction to YOLOv8 model

**Fig 1:**
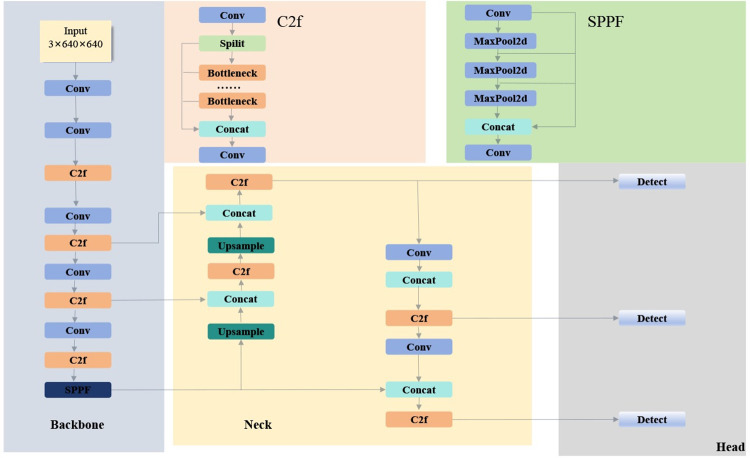
Schematic of the YOLOv8 network structure.

YOLOv8 is a YOLO series object detection algorithm launched by Ultralytics in 2023. It draws on the design advantages of previous YOLO models and makes comprehensive improvements based on the YOLOv5 model structure. YOLOv8 is categorized into five specification scales: n, s, m, l, and x. These scales indicate a progression in complexity and, correspondingly, an increase in accuracy. This paper focuses on YOLOv8n as the baseline model. Distinguished by its accelerated processing speed and reduced parameter count, YOLOv8n is deemed especially apt for integration into underwater detection apparatus. The architecture of this network is depicted in **Fig 1**.

Following the architectural legacy established by earlier models in the YOLO series, the YOLOv8 network is meticulously organized into four principal components: Input, Backbone, Neck, and Head.

Input: To enrich the dataset and enhance the generalization capability of the model, YOLOv8 employs a series of data augmentation techniques, including Mosaic, Mixup, random perspective transformation, and HSV augmentation. Additionally, the input images undergo meticulous preprocessing, which includes dynamically resizing them to a fixed size, performing normalization, and converting the color space. These measures significantly improve the model’s performance and robustness.

Backbone: The backbone network of YOLOv8 is ingeniously constructed with three pivotal modules: Conv, C2f, and SPPF. The Conv module is richly equipped with convolutional layers, batch normalization, and SiLU activation functions. A noteworthy advancement in YOLOv8 is the resizing of the first convolution layer’s kernel from 6x6 to 3x3, aiming for enhanced precision and efficiency. Drawing inspiration from YOLOv7’s C3 module and the ELAN concept, the C2f module innovatively incorporates additional skip-layer connections and Split operations. This design choice enhances YOLOv8’s ability to capture an enriched gradient flow, while ingeniously maintaining a lean structural footprint. The SPPF module, consistent with its predecessors, utilizes spatial pyramid pooling to adeptly handle images of varying sizes. This empowers the model to recognize objects across different scales with finesse, further solidifying its robustness and adaptability in diverse detection scenarios.

Neck: In comparison to the YOLOv5 model, the Neck section removes two convolutional connection layers preceding the upsampling process, rendering the model more streamlined. It incorporates strategies such as Feature Pyramid Networks and Path Aggregation Network (FPN+PAN) [32, 33], utilizing both top-down and bottom-up approaches. This cross-layer connection architecture merges deep and shallow semantic information, enabling the model to combine diverse features for more effective detection of objects varying in size.

Head: A transformative shift in the Head component is evidenced by its transition from a traditional coupled head to a decoupled head architecture. This innovative change distinctly segregates the tasks of classification and detection, addressing the unique demands of classification accuracy and localization precision. Additionally, the model adopts an Anchor-Free approach instead of the conventional Anchor-Based system, marking a strategic pivot designed to enhance detection dynamics. This modification notably boosts the detection speed, reflecting an evolution in the model’s performance capabilities, thereby improving its efficiency and responsiveness in real-time detection scenarios.

### 2.2 Introduction to the dataset

#### 2.2.1 The dataset of URPC2020

The URPC2020 dataset (http://www.urpc.org.cn/index.html) is a widely recognized collection used in China’s underwater robotics competitions throughout the years, encompassing 5543 images that showcase four object types: holothurian, echinus, scallop, and starfish. In our methodology, we divided the dataset into training, test, and validation sets using a 7:2:1 ratio, resulting in a distribution of 3880 images for training, 554 images for validation, and 1109 images for testing. The advantage of this division is to ensure that there is enough data to provide the model with better training, while effectively using the validation set to evaluate model performance and avoid overfitting, and using 20% of the test set to assess model performance in a more neutral and objective manner. The finalized dataset, URPC2020_1, has been made publicly available on Figshare.

#### 2.2.2 The dataset of DUO

**Fig 2:**
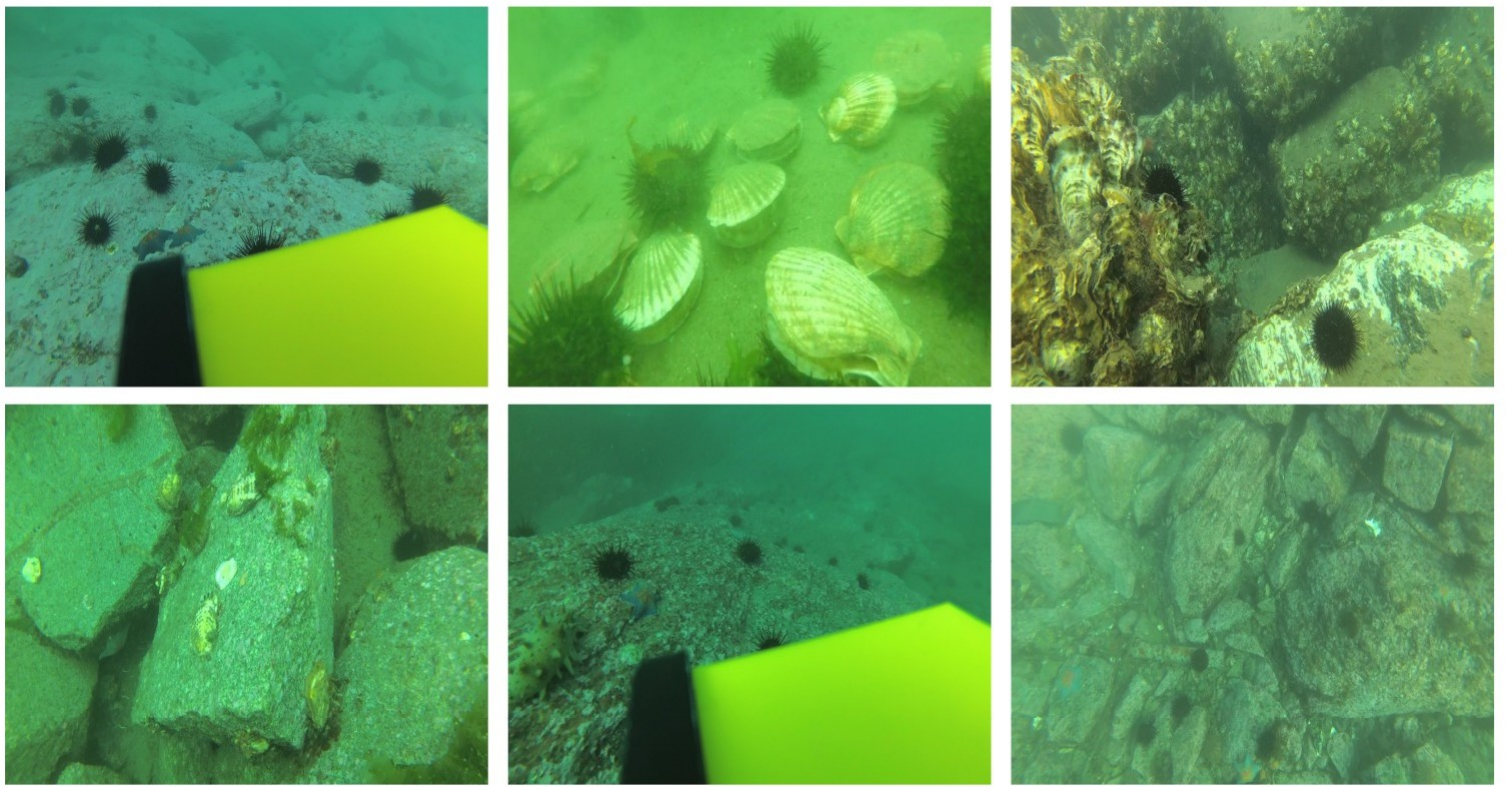
Some images from the dataset.

The DUO dataset [34] (https://github.com/chongweiliu) is a collection of data compiled from URPC competitions over the years, from which duplicates were removed and labels were updated. We reorganized the DUO dataset, resulting in a total of 5208 images, and **Fig 2** presents a selection of images from this dataset. The finalized dataset, DUO_1, has been made publicly available on Figshare.

**Fig 3:**
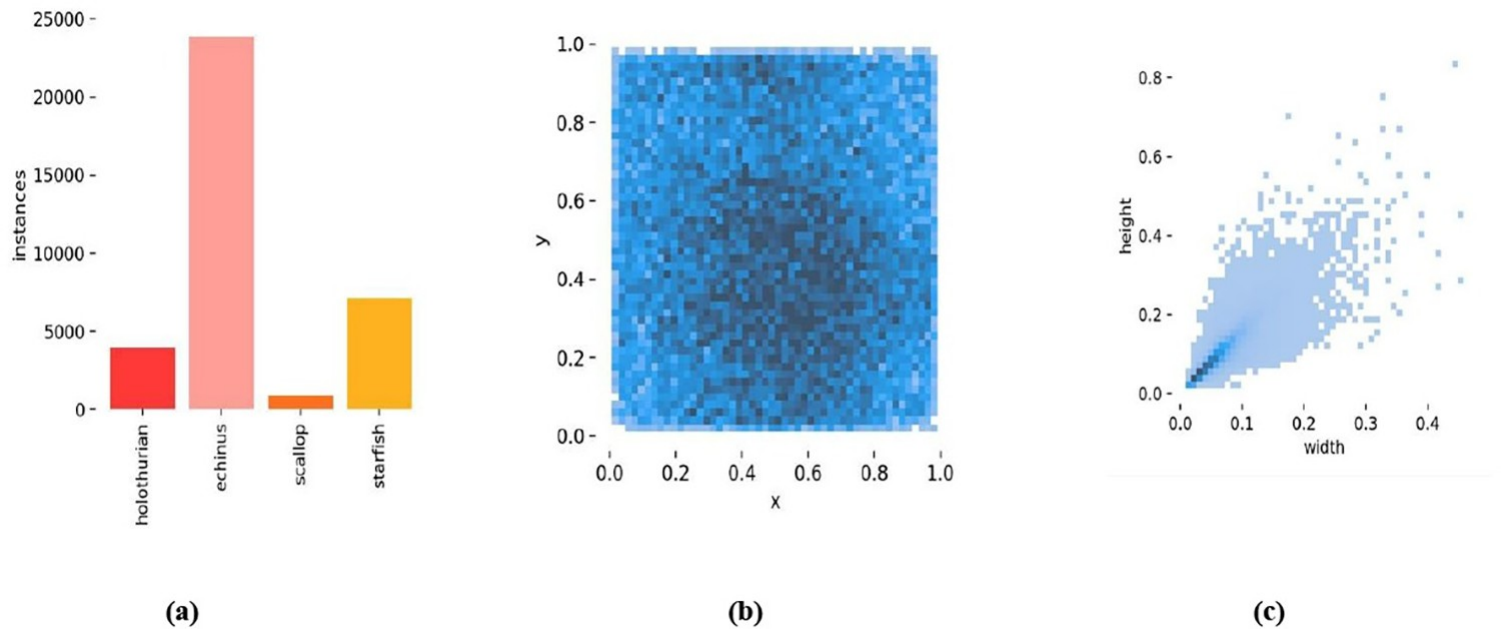
Label information of the dataset. (a) The number of samples in each category (b) The position information of the object center point relative to the entire picture (c) The size ratio of the object relative to the picture.

The dataset encompasses four distinct categories of underwater objects: holothurian, echinus, scallop, and starfish. The dataset was divided into three subsets: training, validation, and test. This resulted in 3645 training images,1043 test images,and 520 images for validation. The ratio of the three sets was 7:2:1, **Fig 3** illustrates the specifics of the dataset labeling. **Fig 3(A)** displays the sample distribution per category, highlighting that echinus has the most samples, while scallop has the fewest samples. **Fig 3(B)** visualizes the positional information of the objects, with denser dots indicating higher object concentrations. this reveals that most objects are situated near the center of the images. **Fig 3(C)** portrays the size distribution of the objects, indicating that the majority are small and clustered in the bottom-left corner of the plot.

## 3. Methods

**Fig 4:**
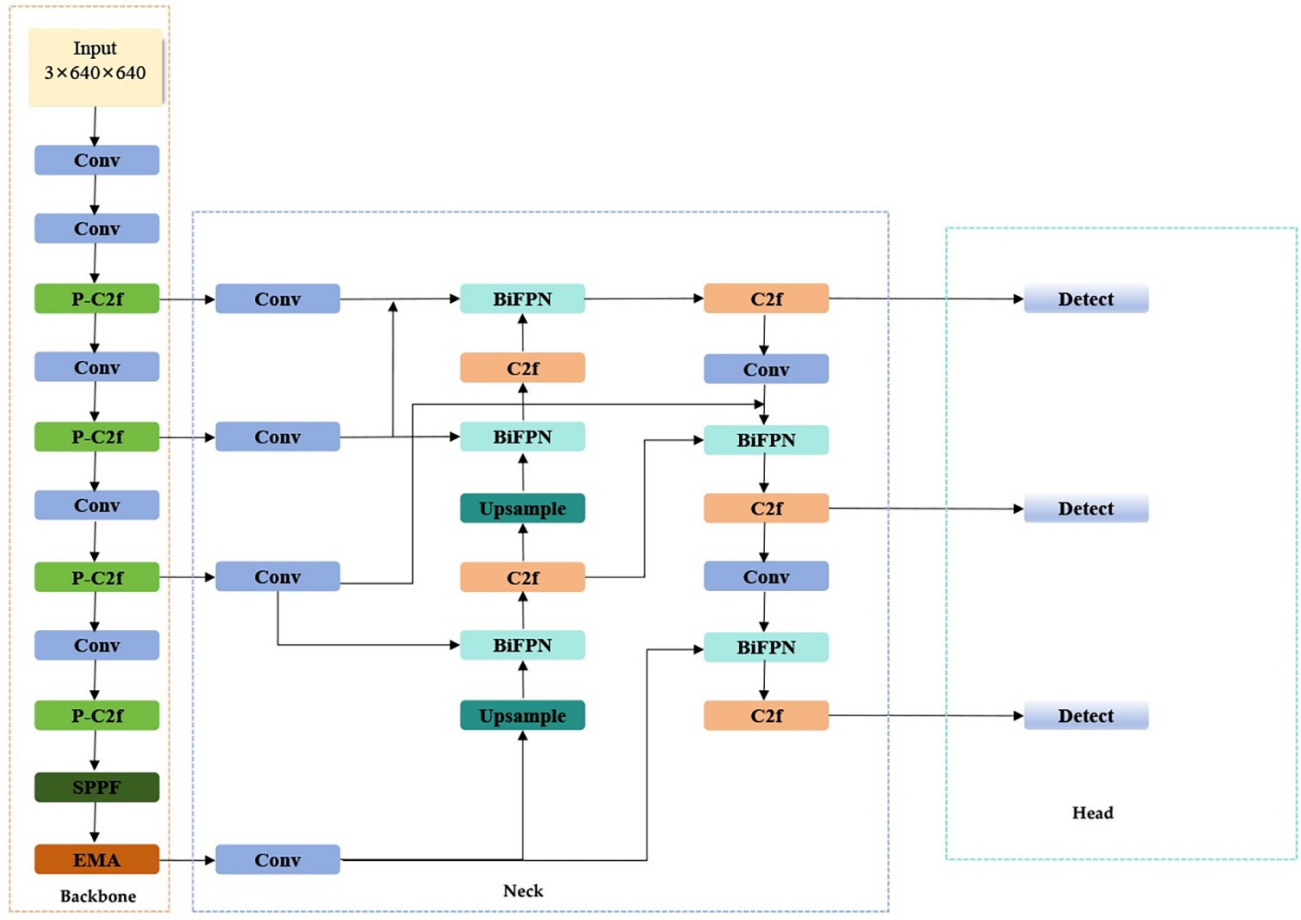
The overall framework of FEB-YOLOv8.

With the goal of achieving a more streamlined network model suitable for deployment on underwater detection equipment, we present the innovative FEB-YOLOv8 network model. This newly proposed model is designed with an emphasis on compactness and efficiency without compromising performance. The overarching framework of FEB-YOLOv8 is systematically illustrated in **Fig 4**.

Our model, FEB-YOLOv8, is optimized from several perspectives—namely the feature extraction network, attention mechanism, and multi-scale feature fusion—to develop a highly effective detection model. The optimization strategy unfolds as follows:

1. Given that the C2F module incorporates multi-layer convolution and pooling operations, this naturally increases the network’s training complexity, necessitating additional computational resources and extended training durations. To address these challenges, we integrate the concept of PConv from FasterNet, substituting the traditional Bottleneck in the C2F module with a FasterNet Block, thereby creating the P-C2F (PConv-C2F) module. This adaptation significantly reduces computational demands and enhances memory access speed, streamlining the overall training process.
2. To compensate for the potential accuracy loss caused by the substitution of PConv, the FEB-YOLOv8 model integrates the EMA module within its backbone network. It enhances model accuracy by facilitating more nuanced feature integration and providing objected attention guidance, thereby ensuring the model’s performance remains uncompromised despite its lightweight architecture.
3. Building upon the feature fusion network of FPN-PAN and drawing inspiration from BiFPN, we design a novel feature pyramid network structure. This structure introduces the P2 feature map information, incorporates cross-scale connection methods, and employs a weighted feature fusion approach. These enhancements enable the model to amalgamate a broader range of features without significantly escalating computational costs. As a result, the model’s complexity is reduced while simultaneously improving its detection accuracy, striking an optimal balance between performance and resource efficiency.

### 3.1 Backbone network improvements

YOLOv8 retains the feature extraction network framework of YOLOv5, with a significant modification, namely, the transition from the C3 module to the C2f module. This change enriches the network model by incorporating additional skip-layer connections and Split operations within the C2f module, enabling the acquisition of more comprehensive gradient flow information. Consequently, this enhances the model’s accuracy. Nonetheless, the C2f module’s extensive use of convolution operations leads to an increase in model parameters, which, in turn escalates the resources and time required for training. To mitigate these challenges, we adopt the innovative concept of PConv, as proposed by Chen et al. in the FasterNet network. By replacing some of the standard convolutions in the C2f module with PConv, we effectively streamline the model’s complexity, striking a balance between efficiency and performance.

**Fig 5:**
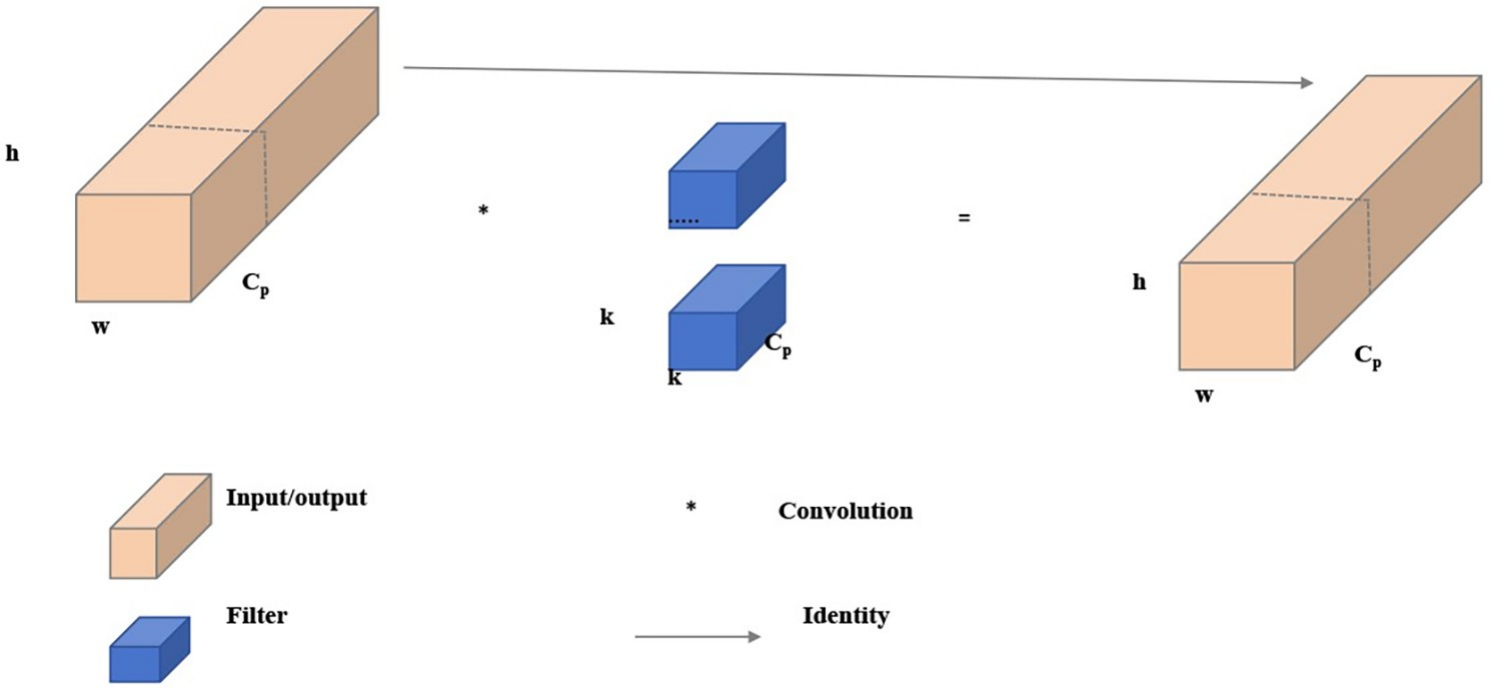
The network structure of Pconv.

In previous lightweight networks like MobileNet [35], ShuffleNet [36], and GhostNet [37], Depthwise Convolution (DWConv) and Group Convolution (GConv) are typically employed. Compared to traditional convolution operations, DWConv applies filters to each input channel individually to generate corresponding outputs, thereby reducing computational demands. However, the exclusive use of DWConv often leads to accuracy loss in the network. To compensate, practitioners tend to increase the number of channels in the network, such as expanding the network width to six times its original size. While this method can boost accuracy, it also escalates memory access requirements, which can lead to increased latency, particularly on devices with memory constraints. To address these dual challenges of computational redundancy and memory access, Chen et al. proposed the Partial Convolution (PConv) technique, as illustrated in **Fig 5**. PConv performs regular convolution operations on only a subset of the input channels C_p_, leaving the remaining channels unchanged, with the idea that the result obtained in this way represents the computation of the entire input feature map. Consequently, this approach significantly reduces computational complexity and is far less computationally intensive than conventional convolution.

Formulas (1) and (2) represent the calculations for the Floating Point Operations (FLOPs) of Conv and PConv, respectively.


$$FLOPs_{Conv}=h\times w\times k^{2}\times c^{2}$$ (1)

$$FLOPs_{PConv}=h\times w\times k^{2}\times c_{p}^{2}$$ (2)

As indicated by the formula, for a feature map with dimensions h×w×c, if the number of partial input channels C_p_ is set to 1/4 of the output feature channel number c, the FLOPs required for PConv amount to merely 1/16 of those necessary for a conventional Convolution. This substantial reduction in computational requirements ensures that the network can maintain its accuracy while simultaneously alleviating the computational and memory burden. This efficiency is crucial for deploying sophisticated models on devices with limited computational resources.

**Fig 6:**
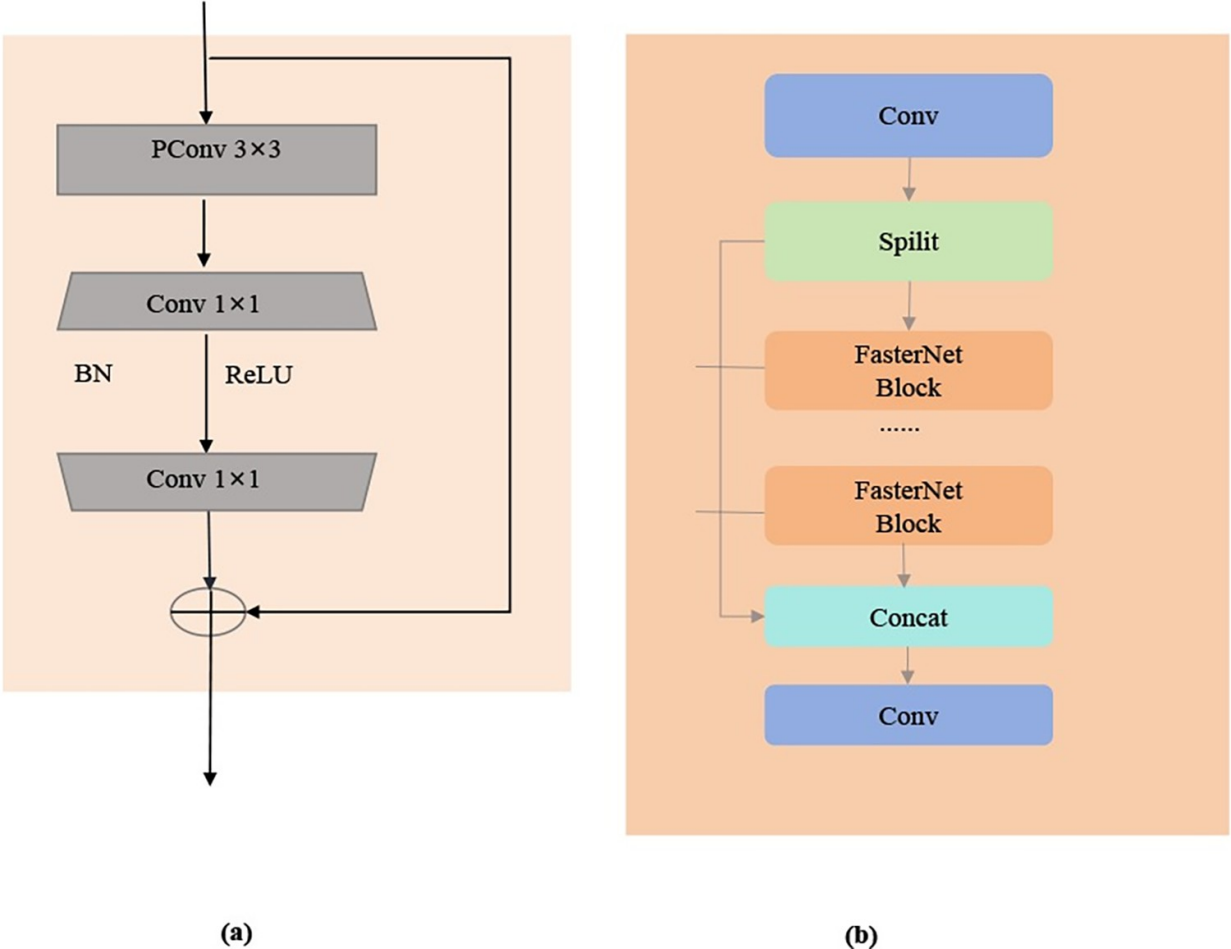
Overall structure of FasterNet Block and P-C2F. (a) FasterNet Block, (b) P-C2f.

Building on the PConv concept, Chen et al. developed the innovative FasterNet Block module, as showcased in **Fig 6(A)**. This advanced module is constructed with a central PConv layer bracketed by two streamlined 1×1 convolutional layers. Such a structure greatly simplifies the model’s complexity while expediting the detection process. In our work, we put forward the P-C2f module, which is an iteration of the FasterNet Block. This module supersedes the Bottleneck in the C2f module, as demonstrated in **Fig 6(B)**.

### 3.2 EMA (Efficient Multi-Scale Attention) module

Attentional mechanisms have come to play an increasing role in computer vision, enabling models to pay more attention to key features in an image, thereby ignoring unimportant information for accurate localization, and are particularly effective in dense, occluded environments. We compare some of the currently used attention modules, including EMA, Coordinate Attention (CA) [38], Squeeze-and-Excitation (SE) [39], A Simple,Parameter-Free Attention Module (SimAM) [40] and Mixed Local Channel Attention (MLCA) [41], and through comparison we find that the EMA module is the most effective, with significant enhancement to the model, and is remarkably adept at addressing the challenges associated with detecting objects within the complex and dynamic underwater environments.

**Fig 7:**
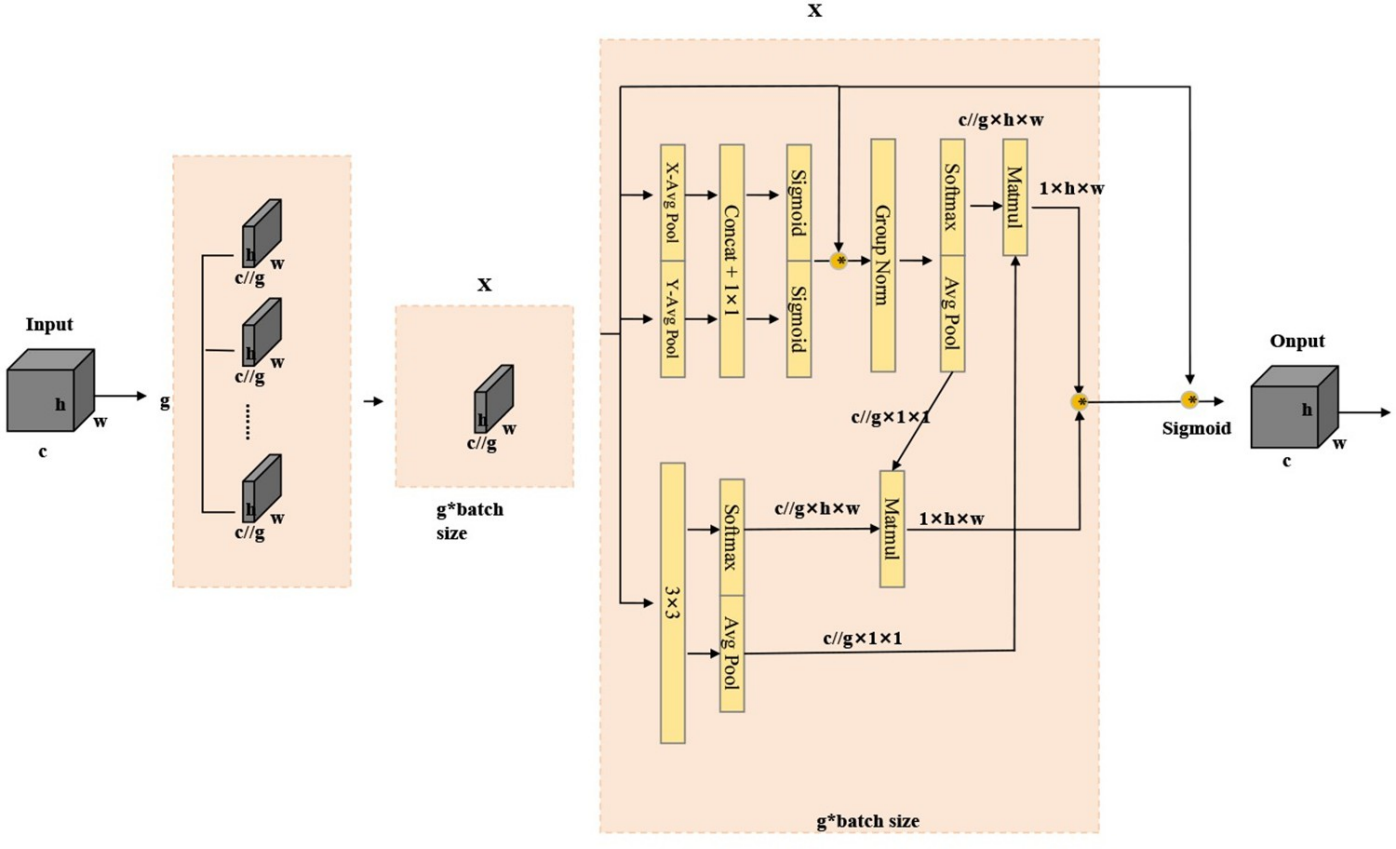
The overall architecture of EMA. "g" = devided grops,"X avg Pool" = 1D horizontal global pooling, "Y avg Pool" = 1D vertical global pooling.


Traditional attention mechanisms typically compress the channel dimension through pooling or similar methods to decrease computational demands. However, this method risks losing critical information. The EMA module adopts an innovative strategy by reconfiguring a segment of the channel dimension into a bulk dimension, thereby mitigating computational load while preserving information integrity. It employs a multi-scale parallel network architecture that bolsters the network’s feature extraction prowess. This is achieved by capturing features at different scales through 1x1 and 3x3 branches, which help establish both short-term and long-term dependencies. Outputs from these parallel branches are then amalgamated through cross-dimensional interactions, augmenting the network’s capacity to discern spatial visual information. This improvement allows the network to achieve a more comprehensive understanding and representation of the input images. The architecture of the EMA module is illustrated in **Fig 7**.

$$z_{c}^{H}(H)=\frac{1}{W}\sum_{0\leq i\leq W}x_{c}(H,i)$$ (3)

$$z_{c}^{W}(W)=\frac{1}{H}\sum_{0\leq j\leq H}x_{c}(j,W)$$ (4)

Subsequently, the feature maps derived from both the height and width directions undergo a concatenation operation. This operation is followed by feature transformations achieved through the Sigmoid activation function. The result in the joining of the two spatial attention maps through a direct element-wise multiplication, enabling cross-channel interaction. In parallel to these operations, the 3×3 branch employs a 3×3 convolutional kernel to extract features, thereby enriching the network’s capacity to process and interpret the input data across various scales and dimensions. This dual-branch approach effectively integrates fine-grained and broader contextual information within the network.

Within the cross-space learning module, 2D global average pooling is performed on the outputs of both the 1×1 and 3×3 branches. Following this, the Softmax function is applied to execute a linear transformation, resulting in the production of two spatial attention maps. The final step involves aggregating the weight values from these two spatial attention maps to generate the output values. Formula (5) represents 2D global average pooling.


$$z_{c}=\frac{1}{H\times W}\sum_{j}^{H}\sum_{i}^{W}x_{c}(i,j)$$ (5)

Given the intricate nature of underwater environments and the prevalence of numerous small and densely packed objects, challenges such as missed detections and inaccuracies frequently arise in model performance concerning underwater objects. To address these issues, the EMA module has been incorporated into the backbone network. This strategic inclusion aims to enhance the network’s focus on multi-scale information, thereby bolstering its feature extraction capabilities. As a result, this enhancement significantly improves the model’s accuracy, making it more proficient at identifying and accurately classifying underwater objects amidst the complexities of their surroundings

### 3.3 Feature pyramid optimization

First introduced into the YOLOv3 network, the Feature Pyramid Network (FPN) is a ground-breaking bottom-up feature transfer mechanism. This approach facilitates the conveyance of deep-layer information to the more superficial layers, thereby enriching the semantic depth of the network’s representations. However, this advancement had somewhat limited localization capabilities. To counterbalance this limitation, the subsequent YOLOv4 network introduced the Path Aggregation Network (PAN), which supplements the FPN framework with a top-down feature delivery mechanism. This improvement greatly improves the model’s learning and accurate object localization performance, addressing this limitation. Following this innovation, all subsequent feature fusion networks have utilized the FPN-PAN combination to optimize performance. To enhance simplicity and efficiency, YOLOv8 further refined this architecture by eliminating nodes that had not undergone feature fusion, resulting in a more streamlined network structure.

**Fig 8:**
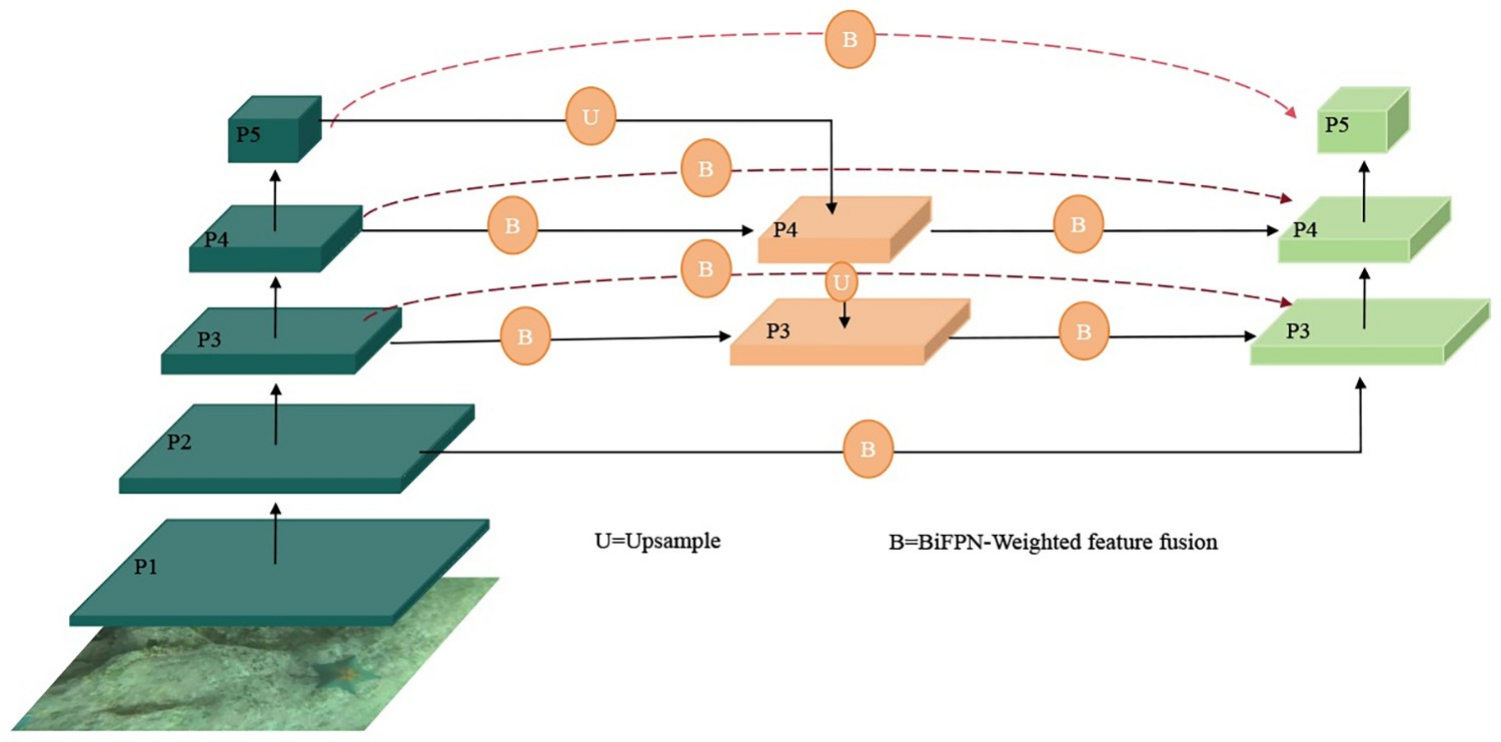
Feature pyramid optimization.

Despite its significant success, the FPN-PAN architecture still falls short in detecting tiny objects is still not up to par. To address this shortcoming, our paper introduces the Bidirectional Feature Pyramid Network (BiFPN), which improves the network by adding an additional pathway at the same layer between the original input and output nodes and removing nodes that do not contribute to effective feature fusion. Furthermore, an effective weighted feature fusion mechanism is employed instead of the original simple feature summation approach. Given that underwater objects are predominantly small and prone to feature loss during deep network processing—which can lead to potential non-detection—we have integrated P2 feature map information with the original BiFPN. We also include additional convolution to modify image size and establish skip connections between the original input node and the output node at the same level, applying a weighted feature fusion approach. This is depicted in **Fig 8**. The benefit of this innovation is the network’s augmented capacity to fuse information across multiple scales, thereby improving detection capabilities without incurring significant additional computational costs.

## 4. Experiment

### 4.1 Experimental environment and configuration

For this experiment, we set up the required environment on a computer running the Ubuntu operating system, as detailed in **Table 1**. Python 3.8 was used as the programming language for the project, while PyTorch 2.0.0 was used as the deep learning framework. The CUDA version installed was 11.8. In terms of hardware, the computer was equipped with an AMD EPYC 7742 CPU and an NVIDIA GeForce RTX 3090 GPU with 24GB of video memory. This configuration provided the necessary computational power and memory capacity to effectively run and evaluate our proposed network model.

**Table 1:** Experimental environment.

Parameters	Configuration
CPU	AMD EPYC 7742
GPU	NVIDIA GeForce RTX 3090
GPU memory size	24GB
Operating systems	Ubuntu 18.04
Python	Python 3.8
Framework	Pytorch 2.0.0
CUDA	11.8

Key parameter configurations employed during the training phase are as detailed in **Table 2**. Input images were standardized to 640×640 pixels, with the Mosaic data augmentation technique applied to enhance model robustness. Training spanned 300 epochs. Momentum was set to 0.937, and both the initial and final learning rates were fixed at 0.01. The weight decay parameter was set to 0.0005 to help avoid overfitting. Stochastic Gradient Descent (SGD) algorithm was used for model optimization, ensuring efficient and effective convergence to the optimal solution. Batch size was set to 16.

**Table 2:** Parameters required for model training.

Parameters	Setup
Epochs	300
Momentum	0.937
Initial learning rate	0.01
Final learning rate	0.01
Weight decay	0.0005
Batch size	16
Input image size	640×640
Optimizer	SGD
Data enhancement strategy	Mosaic

### 4.2 Model evaluation metrics

To evaluate the performance of the FEB-YOLOv8 model, we used several widely recognized metrics: Precision (P), Recall (R), Average Precision (AP), and Mean Average Precision (mAP). The mAP metric is further subdivided into mAP@0.5 and mAP@0.5:0.95, reflecting the model’s average accuracy across all categories at IoU thresholds of 0.5 and from 0.5 to 0.95, respectively. Additionally, we evaluated the model’s parameters (Parameters), Giga Floating-point Operations Per Second (GFLOPs), Frames Per Second (FPS), and Model Size as crucial performance indicators for this experiment.

Precision (P) measures the percentage of samples predicted as positive by the model that are actually positive. Recall (R) measures the percentage of true positive samples that are correctly identified by the model out of all actual positive samples. Formulas (6) and (7) provide the definitions for Precision and Recall.


$$Precision=\frac{\mathrm{TP}}{\mathrm{TP}+\mathrm{FP}}$$ (6)

$$Recall=\frac{\mathrm{TP}}{\mathrm{TP}+\mathrm{FN}}$$ (7)

In these metrics, TP (True Positive) indicates cases where the model correctly predicts a positive sample as positive. FP (False Positive) represents cases where the model incorrectly classifies a negative sample as positive. FN (False Negative) refers to cases where the model incorrectly classifies a positive sample as negative.

Average Precision (AP) is the area under the Precision-Recall (P-R) curve. It represents the average precision for a single category, as illustrated by Formula (8).


$$AP=\int_{0}^{1}P(R)\mathrm{dR}$$ (8)

Mean Average Precision (mAP) is defined as the mean of Average Precision(AP) values across all categories and is calculated as shown in Formula (9).


$$mAP=\frac{1}{x}\sum_{i=1}^{x}AP_{i}$$ (9)

### 4.3 Experimental results and analysis

#### 4.3.1 Experimental results

**Fig 9:**
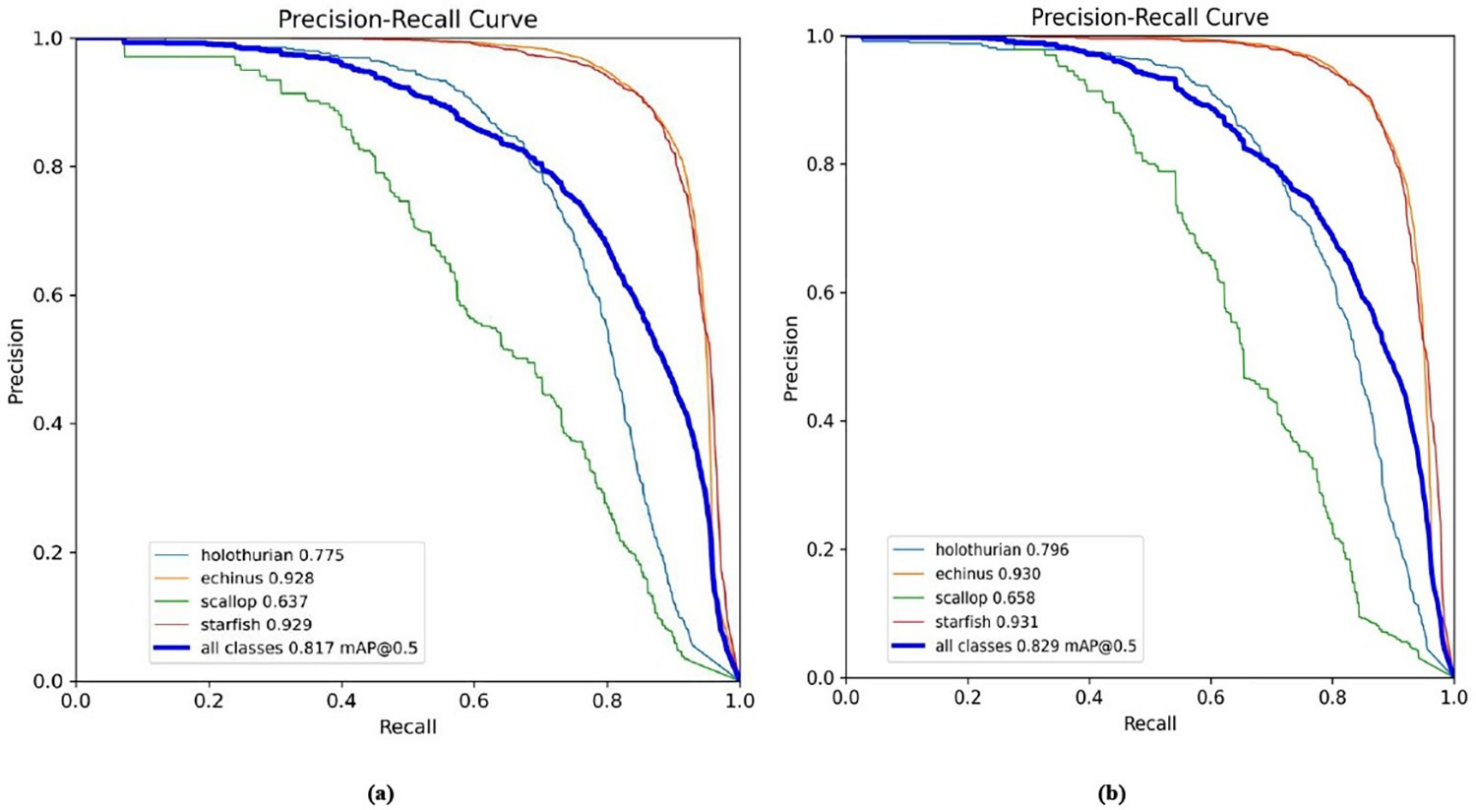
Comparison between YOLOv8 model and FEB-YOLOv8 model. (a) YOLOv8 (b) FEB-YOLOv8.

We performed a comparative performance evaluation of the YOLOv8 model and our newly developed FEB-YOLOv8 model on the DUO dataset, as shown in **Fig 9**. The analysis reveals that our FEB-YOLOv8 model has significantly improved detection efficiency across various categories. Notably, in the "holothurian" category, the AP increased by 2.1%. The mAP value also reached 82.9%, an improvement of 1.2%. This improvement demonstrates the FEB-YOLOv8 model’s superior performance over the original YOLOv8 model and emphasizes how well it addresses the difficulties associated with underwater object detection.

#### 4.3.2 Comparison with different models

We performed a thorough comparison with the state-of-the-art deep learning object detection models, including SSD, Faster R-CNN, YOLOv3, YOLOv4, YOLOv7-Tiny, and YOLOv10n in the DUO dataset, to demonstrate the superior performance of the FEB-YOLOv8 model. The evaluation metrics employed for this comparison were mAP@0.5, mAP@0.5:0.95, Parameters, GFLOPs, and FPS. These metrics collectively offer insights into each model’s accuracy, computational efficiency, and speed. **Table 3** presents the comprehensive findings of this comparative analysis, providing a clear overview of how the FEB-YOLOv8 model stands in relation to its contemporaries in the field of object detection.

**Table 3:** Comparison of different object detection models on the DUO dataset.

Model	mAP@0.5/%	mAP@0.5:0.95/%	Parameters/M	GFLOPs/G	FPS
SSD	79.7	50.7	24.01	274.4	70.6
Fatster R-CNN	74.4	39.3	136.75	401.7	38.2
YOLOv3	71.6	40.3	61.54	155.3	69.1
YOLOv4	76.7	43.9	63.95	141.9	51.9
YOLOv7-Tiny	81.0	57.7	6.02	13.2	105.2
YOLOv8n	81.7	61.8	3.01	8.2	140.8
FEB-YOLOv8	82.9	63.2	1.64	6.2	116.2
YOLOv10n	81.9	61.7	2.70	8.4	112.3

According to the data presented in **Table 3**, our newly developed FEB-YOLO model surpasses other object detection models in both accuracy and efficiency. Specifically, in terms of accuracy, the FEB-YOLO model exhibits a notable improvement over the YOLOv8n model, with an increase of 1.2% in mAP@0.5 and 1.4% in mAP@0.5:0.95, respectively, When compared with the latest YOLOv10n, the FEB-YOLO model still holds a significant advantage with improvements of 1.0% in mAP@0.5 and 1.5% in mAP@0.5:0.95, respectively. Regarding its lightweight design, the FEB-YOLO model boasts a mere 1.64M parameters and requires only 6.2G of computational operations. Although there is a slight reduction in FPS compared to YOLOv8, due to the addition of more convolutional operations which increase the model’s computational demand, but it still exceeds other object detection models and meets the requirements of real-time detection.

To rigorously assess the model’s generalizability, we conducted comparative experiments on the URPC2020 dataset using the same evaluation metrics and experimental environment. The results of the experiments are shown in **Table 4**.

**Table 4:** Comparison of different object detection models on the URPC2020 dataset.

Model	mAP@0.5/%	mAP@0.5:0.95/%	Parameters/M	GFLOPs/G	FPS
SSD	76.2	37.6	24.01	274.4	70.6
YOLOv3	72.9	31.4	61.54	155.3	69.1
YOLOv4	75.4	34.0	63.95	141.9	51.9
YOLOv5s	80.44	43.5	7.27	17.1	103.1
YOLOvX	79.7	42.0	5.03	15.2	102.7
YOLOv8n	82.2	47.9	3.01	8.2	140.8
FEB-YOLOv8	83.5	48.9	1.64	6.2	116.2

As illustrated by the data in **Table 4**, our proposed FEB-YOLOv8 model achieved 83.5% mAP0.5 and 48.9% mAP0.5:0.95. In comparison to the baseline YOLOv8n model, there was a notable increase of 1.3% in mAP@0.5 and a 1.0% rise in mAP@0.5:0.95, respectively. This exceptional performance not only surpasses that of the current mainstream object detectors but also unequivocally demonstrating the superior generalization capability of FEB-YOLOv8.

#### 4.3.3 Ablation experiments

We performed ablation studies on the DUO dataset for these particular components to verify the effectiveness of the newly added modules within the FEB-YOLOv8 model. Each experiment was initiated under identical conditions, with the parameters detailed in **Table 2**, and without the use of pre-trained weights. The outcomes of these experiments are summarized in **Table 5**. The results indicate that substituting the C2f structure in the backbone network led to a reduction in the number of parameters by 0.36M, a decrease in GFLOPs by 1.1G, and a reduction in model size by 0.7MB, while only minimally impacting accuracy—mAP@0.5 and mAP@0.5:0.95 decreased by 0.1% and 0.2%, respectively. This indicates a leaner model without significant loss in precision. Building on this, the integration of the enhanced BiFPN as the feature fusion network showed further improvements, with an increase of 0.8% in mAP@0.5:0.95 and 0.3% in mAP@0.5 compared to the baseline model. Furthermore, the model size was further reduced by 2.6MB, and there was a noticeable decrease in both the number of parameters and GFLOPs—by 1.38M and 2.0G, respectively. In the final step, incorporating the EMA module into the backbone aimed at compensating for accuracy loss. Compared to the baseline model, the FEB-YOLOv8 algorithm achieved 82.9% mAP0.5 and 63.2% mAP0.5:0.95, representing an increase of 1.2% and 1.4%, respectively. Additionally, the model’s GFLOPs and parameter count have been lowered to 6.2G and 1.64M, respectively, which represents a 45.51% and 24.39% drop from the baseline model, increasing its suitability for tasks involving underwater detection. The performance still satisfies the requirements for real-time detection, even though the FPS is lower than with the baseline model, underscoring the model’s optimized balance between efficiency and effectiveness.

**Table 5:** Ablation experiments.

P-C2f	EMA	BiFPN	mAP@0.5/%	mAP@0.5:0.95/%	Parameters/M	GFLOPs/G	FPS	Model Size/MB
			81.7	61.8	3.01	8.2	140.8	6.0
√			81.6	61.6	2.65	7.1	140.8	5.3
	√		82.3	63.0	3.02	8.3	138.8	6.0
		√	82.5	63.1	1.99	7.2	138.8	4.1
√	√		81.6	62.0	2.66	7.2	111.1	5.3
√		√	82.0	62.6	1.63	6.2	128.2	3.4
	√	√	83.1	63.6	2.0	7.3	123.4	4.1
√	√	√	82.9	63.2	1.64	6.2	116.2	3.4

To validate how distinct attention mechanisms affect the model, we conducted a comparative analysis of a few mainstream attention modules. The details of these comparisons are presented in **Table 6**, which shows the experimental outcomes of incorporating various attention mechanisms into the baseline model.

**Table 6:** Comparison of attention modules.

Model	mAP@0.5/%	mAP@0.5:0.95/%	Parameters/M	GFLOPs/G
YOLOv8	81.7	61.8	3.01	8.2
+EMA	82.3	63.0	3.02	8.3
+CA	81.7	62.6	3.01	8.2
+MLCA	81.9	62.0	3.01	8.2
+SE	82.2	62.0	3.01	8.2
+SimAM	82.2	62.7	3.01	8.2

**Fig 10:**
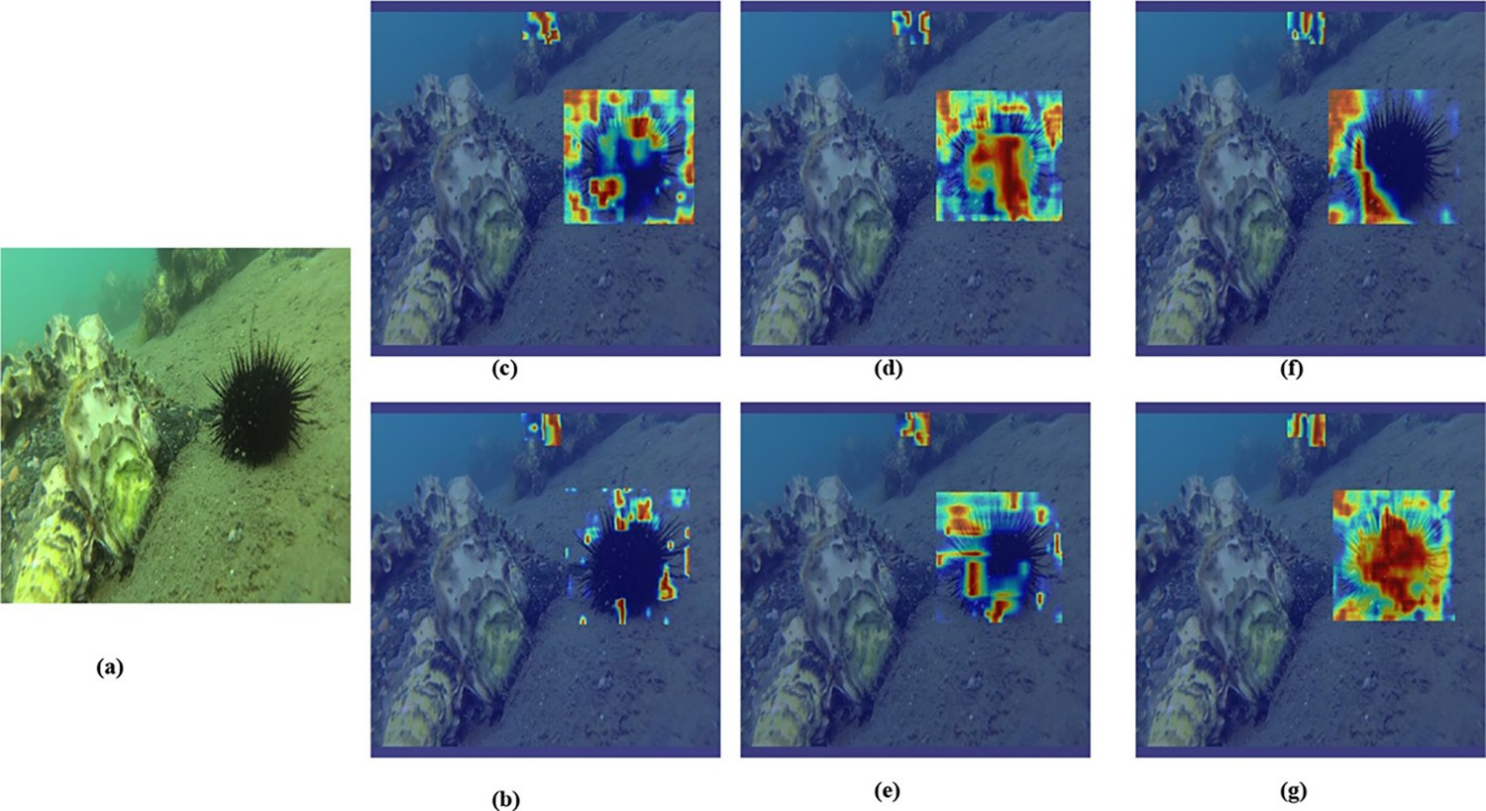
Heat map comparison of adding different attention modules. (a) Original image; (b) YOLOv8; (c) Add SimAM; (d) Add SE; (e) Add MLCA; (f) Add CA; (g) Add EMA.

Each attention module was integrated at the same position within the baseline model, and the results demonstrated that the EMA module provided the most significant improvement, with increases of 0.6% in mAP@0.5 and 1.2% in mAP@0.5:0.95, respectively. To illustrate the enhancement effects of the attention modules, we employed the GradCAM [42] feature map visualization tool. The comparative visualizations, presented in **Fig 10**, show the changes before and after the addition of the attention modules. Specifically, **Fig 10(A)** displays the original image, while **Fig 10(B)** through **Fig 10 (G)** showcase the heat maps for the baseline model and with the addition of the SimAM, SE, MLCA, CA, and EMA modules, respectively. The heat maps reveal a more pronounced thermal signature in the object areas after the incorporation of the EMA module, indicating that it better directs the model’s focus towards relevant features for improved detection capabilities.

**Fig 11:**
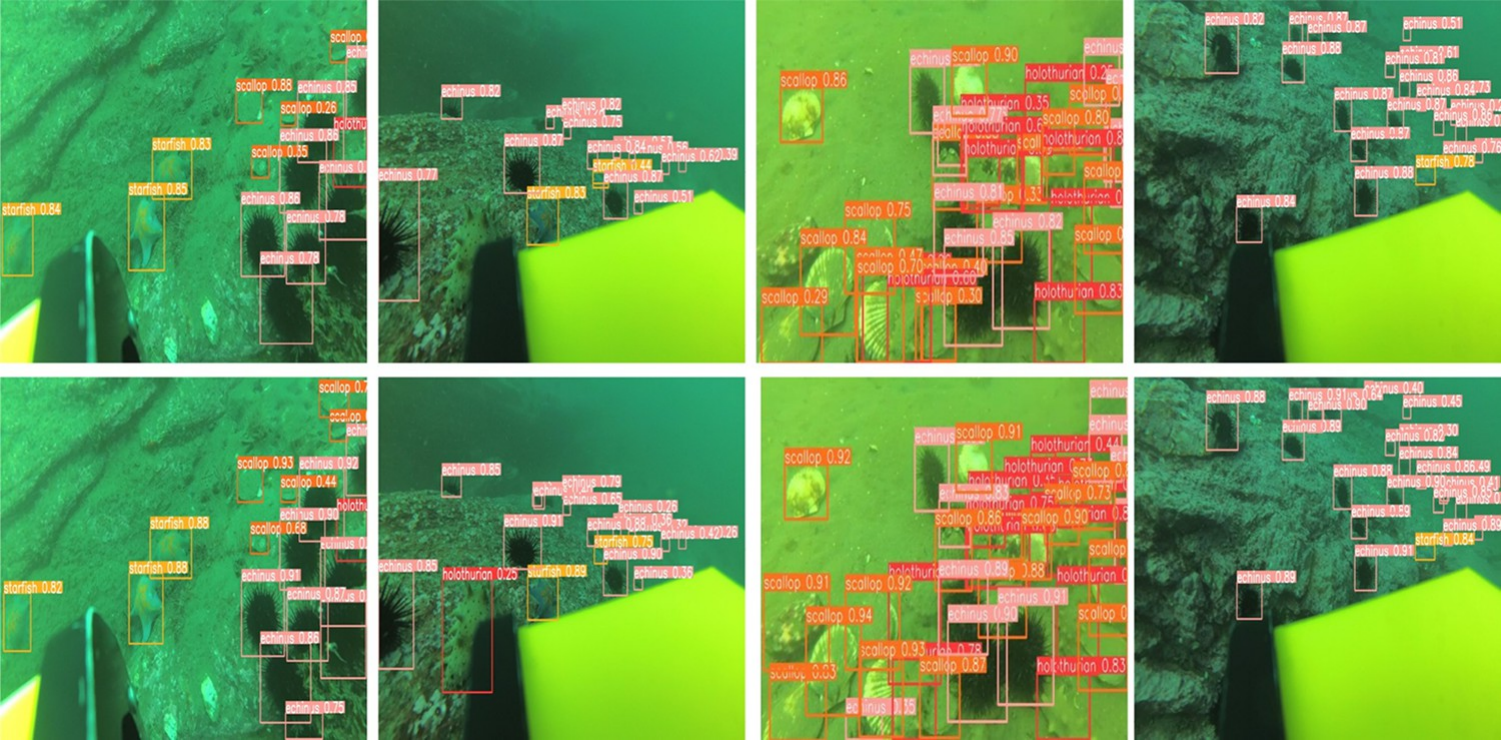
Detection results of YOLOv8 (top) and FEB-YOLOv8 (bottom) in complex environments.

To highlight the superior capabilities of our FEB-YOLOv8 model, we executed a series of tests on several images selected from the test set, as depicted in **Fig 11**. The visual evidence from this figure demonstrates that the FEB-YOLOv8 model excels in detecting smaller objects and achieves greater accuracy in complex scenarios. This performance underscores the model’s enhanced effectiveness in addressing the challenges of object detection with precision.

## 5. Conclusions

To address the challenges of limited storage capacity and computational power in underwater equipment, we enhanced the YOLOv8 model by introducing three modules, culminating in the development of the lightweight underwater model, FEB-YOLOv8. Initially, the FasterNet Block replaced the Bottleneck structure in the backbone network, and the novel P-C2f was introduced, significantly reducing the model’s parameter count and computational load, albeit with a slight compromise in accuracy. Subsequently, the EMA module was added to the backbone structure of the model. This strategic addition aimed to enhance the model’s capability in handling multi-scale information more efficiently, consequently improving its proficiency in identifying small objects within complex settings. Lastly, we integrated the BiFPN feature fusion network, which, through optimization, utilized features from the P2 feature map and added convolutions for image size adjustment, along with skip connections between the initial input and output nodes at corresponding levels. This integration efficiently combines more scale information, enhancing detection capabilities without incurring extra costs.

On the DUO dataset and the URPC2020 dataset, we benchmarked against prevalent object detection algorithms. The experimental findings confirm that our FEB-YOLOv8 model outshines its counterparts in both accuracy and efficiency. While maintaining a low parameter amount (1.64M), low GLOPs (6.2G), and lightweight model size (3.4MB), our model achieves high accuracy. Compared to other underwater object detection models, the FEB-YOLOv8 demonstrates an optimal balance between precision and efficiency, making it exceptionally suitable for underwater detection tasks.

Looking ahead, our model has some limitations. Currently, the underwater datasets are relatively small and of lower quality, which may impact the model’s performance. Our future efforts will focus on collecting larger, higher-quality, and more diverse datasets to improve the model’s scalability and robustness. Additionally, research into data augmentation and pre-processing techniques will be crucial for enhancing dataset quality and further improving the model’s performance.
